# Supplementary figures and images for: Hepatocyte-Specific Fads1 Overexpression Attenuates Western Diet-Induced Metabolic Phenotypes in a Rat Model
Source: Int J Mol Sci. 2024 Apr 29;25(9):4836. doi: 10.3390/ijms25094836 (PMC11084797; doi:10.3390/ijms25094836)

Figure S1

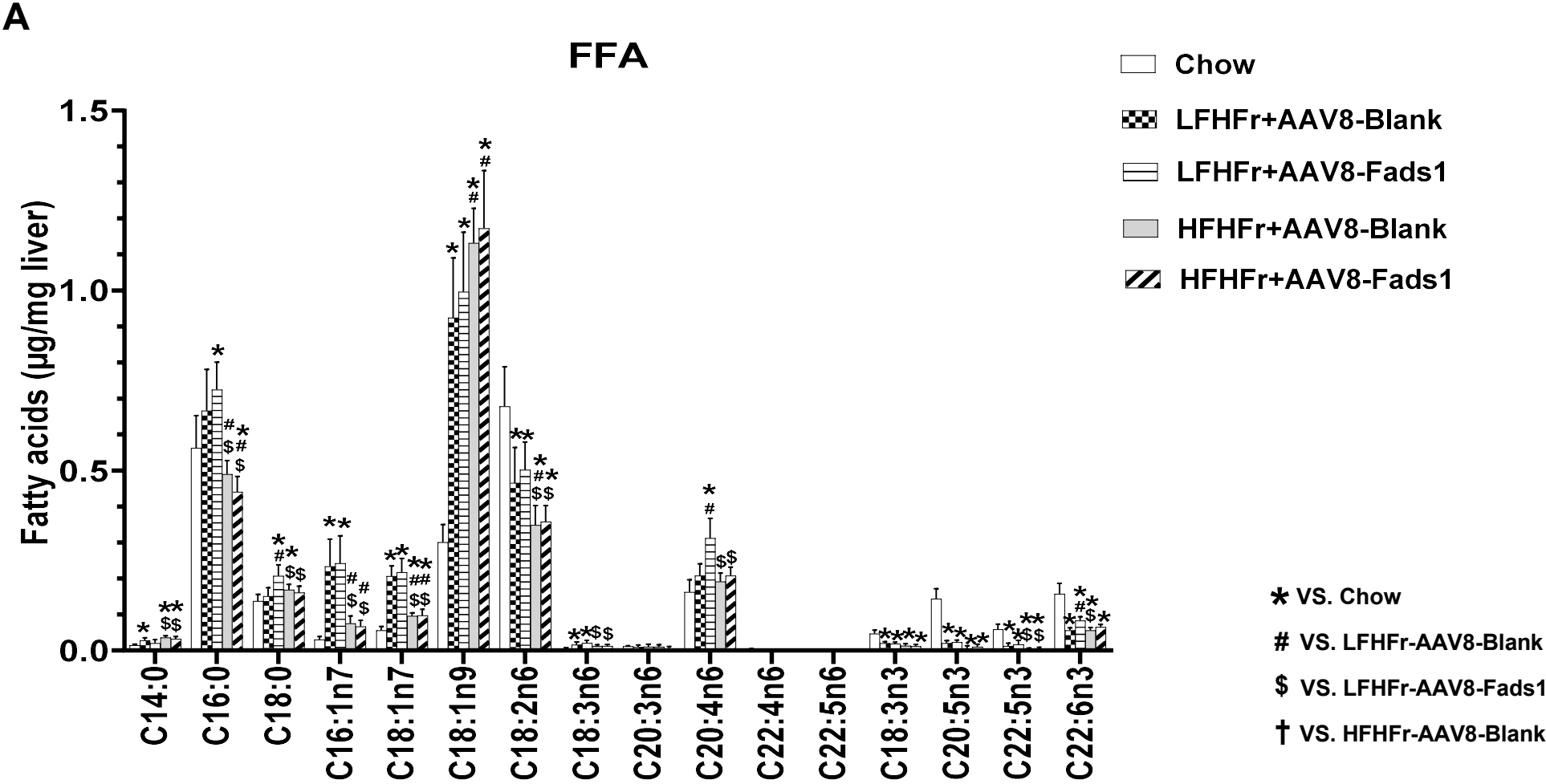

**B****DAG**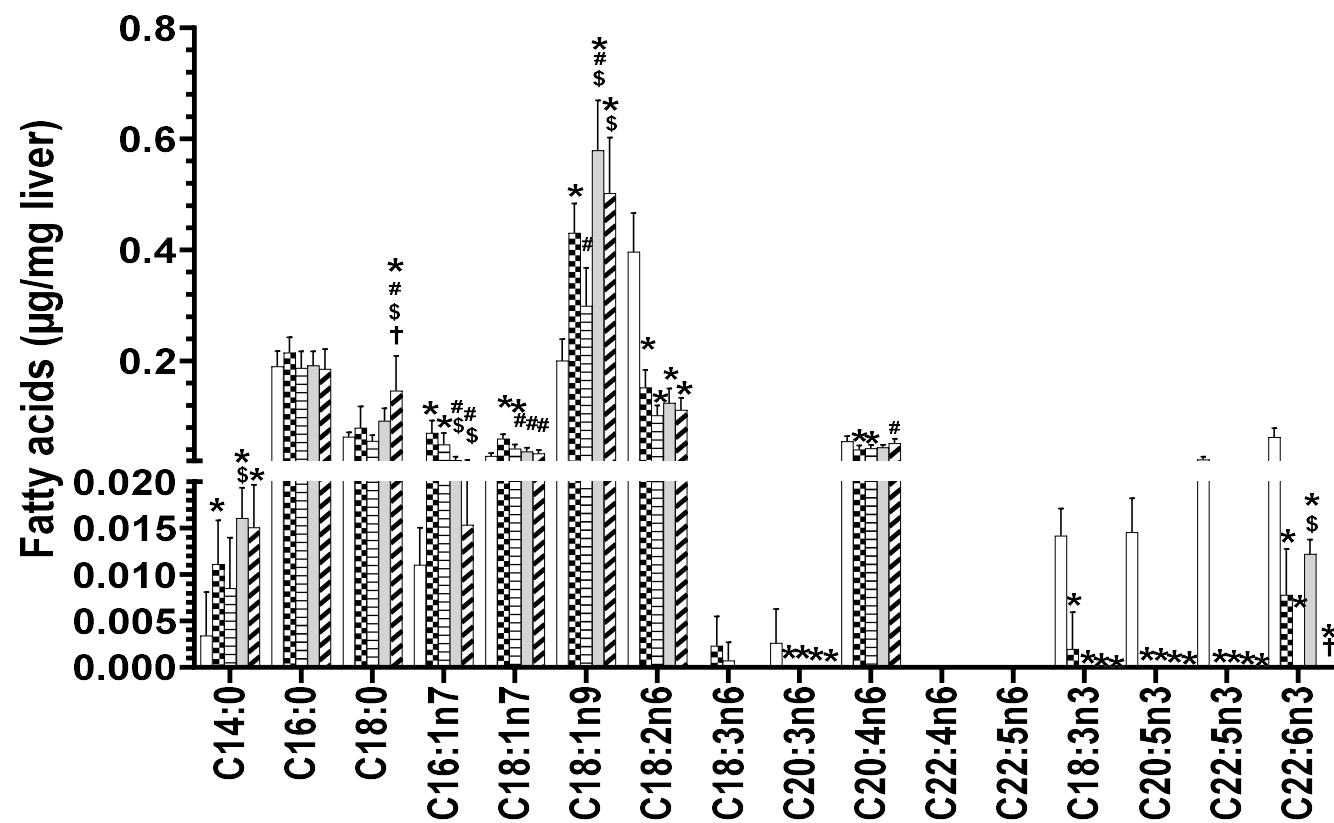

C

TAG

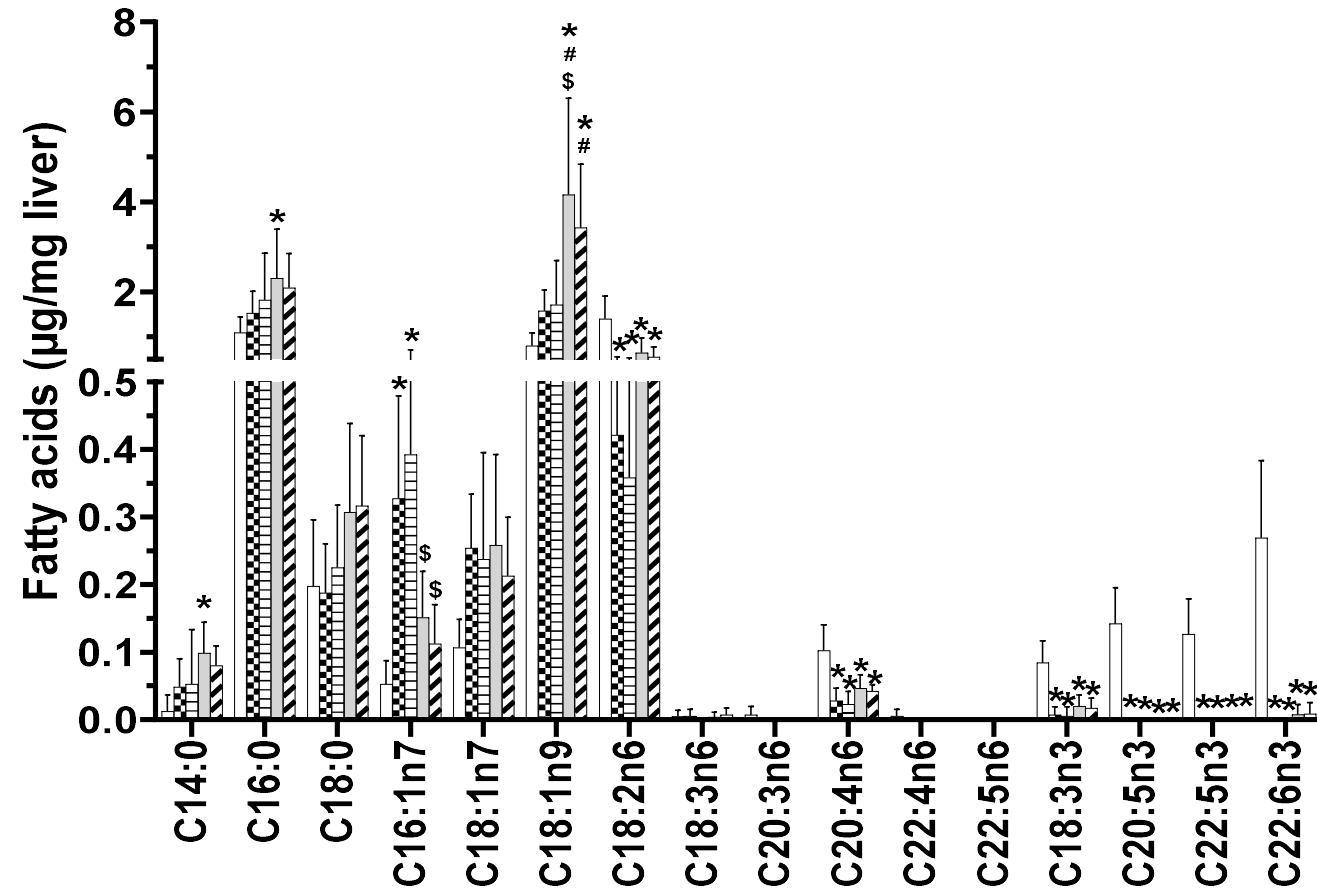

D

PL

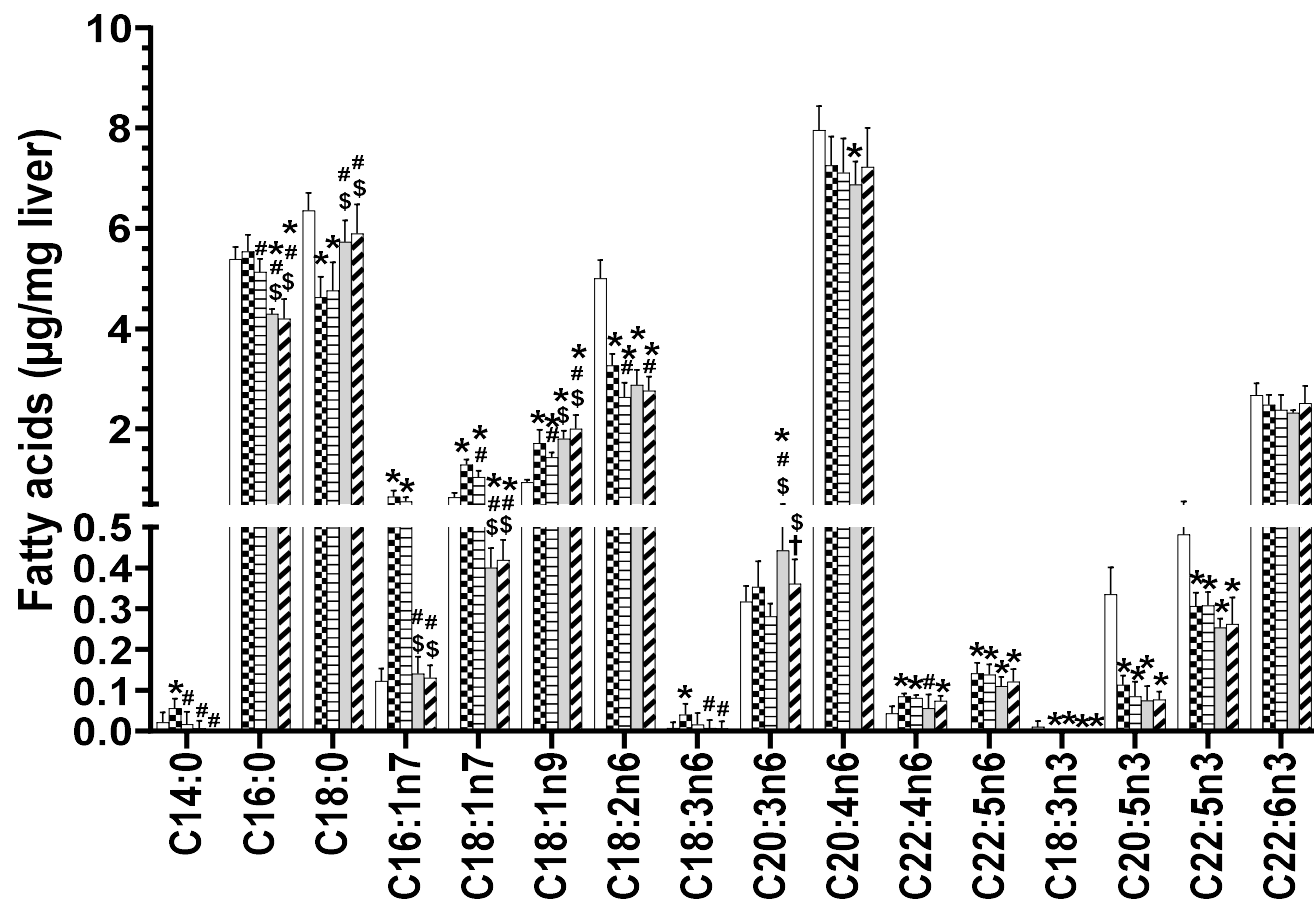

E

CE

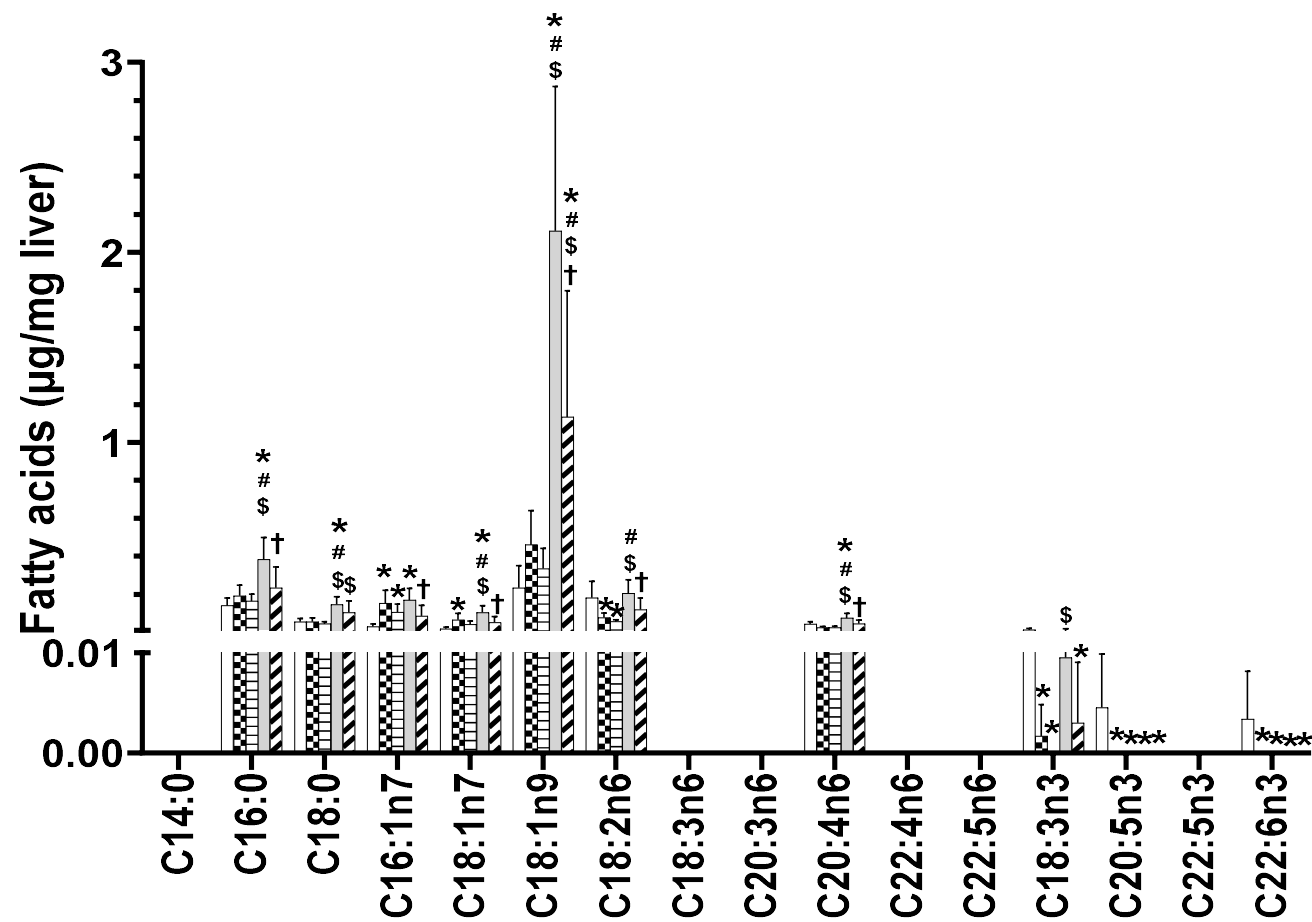

Supplement: Supplementary file 1 [file ijms-25-04836-s001.zip › ijms-2928250-supplementary.pdf]
